# Geographical patterns of the incidence and mortality of colorectal cancer in mainland Portugal municipalities (2007–2011)

**DOI:** 10.1186/s12885-019-5719-9

**Published:** 2019-05-29

**Authors:** R. Roquette, M. Painho, B. Nunes

**Affiliations:** 1 NOVA IMS Information Management School, Universidade Nova de Lisboa, Campus de Campolide, 1099-085 Lisbon, Portugal; 2 Department of Epidemiology, Instituto Nacional de Saúde Dr. Ricardo Jorge, Av. Padre Cruz, 1649-016 Lisbon, Portugal; 3 Centro de Investigação em Saúde Pública, Escola Nacional de Saúde Pública, Universidade NOVA de Lisboa, Av. Padre Cruz, Lisbon, 1600-560 Portugal

**Keywords:** Colorectal cancer, Incidence, Mortality, Geographical distribution, Spatial epidemiology, BYM, GWR

## Abstract

**Background:**

Cancer is a leading cause of morbidity and mortality in the world. In Portugal, colorectal cancer is one of the most incident cancers; thus, it is crucial to act to fight it. Knowledge of the geographical distribution of the incidence and mortality of colorectal cancer can facilitate the execution of these actions and make them more effective.

**Methods:**

Our paper aims to describe and discuss the geographical patterns of colorectal cancer incidence and mortality in mainland Portugal municipalities (2007–2011). We used the Besag, York and Mollié (BYM) model to compute the relative risk (RR) and posterior probability (PP). We performed a cluster analysis with Global Moran’s Index and Local Moran’s Index (LISA). We ran a geographically weighted regression (GWR) to compare incidence and mortality patterns.

**Results:**

Incidence and mortality have different distributions of RR values. The interval of RR concerning incidence was higher than the interval of RR concerning mortality. PP values reinforce the finding of higher heterogeneity of the incidence of colorectal cancer.

The comparison of the cluster maps for incidence and mortality shows a few municipalities classified with the same cluster type in both maps. Additionally, the GWR results show that the percentage of RR mortality explained by RR incidence differs throughout mainland Portugal.

From the comparison of our results with the prevalence of risk factors (at NUTS II level), the need to be aware of smoking habits, alcohol consumption and the unhealthy diet of the Portuguese population stands out.

**Conclusions:**

There are differences in the geographical distribution of the RR incidence and RR mortality of colorectal cancer in mainland Portugal municipalities. Likewise, it is relevant to highlight the cluster of two municipalities with high RR values concerning colorectal cancer’s incidence and mortality. Future research is necessary to explain the geographical differences in the distribution of colorectal cancer in mainland Portugal municipalities. Based on our findings, it may be interesting to examine the influence of smoking, alcohol consumption, diet and screening on colorectal cancer in greater detail. Additionally, it may be relevant to develop an analysis focused on municipalities where the incidence values explain the mortality values poorly (or well).

**Electronic supplementary material:**

The online version of this article (10.1186/s12885-019-5719-9) contains supplementary material, which is available to authorized users.

## Background

Cancer is currently a major public health concern around the world. At the end of the last decade, it was estimated that approximately half of all deaths in Europe in the middle-aged population were due to cancer [1].

Among all cancers, colorectal cancer is the third-most common in the world, and it has a higher incidence in more developed countries [2]. However, mortality by colorectal cancer has been decreasing in the last decades in several European countries, namely, due to improvements in screening and treatments, which promote higher survival rates [3].

In Portugal, cancer mortality has been increasing in the last decades. Between 1960 and 1998, the crude mortality rate has more than doubled [4], and it increased by approximately 3% each year from 2000 to 2005 [5]. Between 2010 and 2014, the crude mortality rate continued to increase, but the standard mortality rate (standardized to the standard European population defined by the World Health Organization) decreased slightly [6], illustrating the effect of population age on the mortality rates. Colorectal cancer accounted for the second-highest standard incidence rate (standardized to the standard European population defined by the World Health Organization) among Portuguese men and women [5]. Therefore, it is important to monitor the evolution of colorectal cancer and establish policies to mitigate the impact of this disease. For this purpose, it is relevant to understand if there are differences in the spatial distribution of colorectal cancer in Portugal.

Another important issue in cancer research is the association between cancer incidence and the occurrence of risk factors. Nevertheless, the long latency period of cancer, that is, the period between the exposure to risk factors and the diagnosis of the disease, hampers the study of these risk factors [7]. Concerning colorectal cancer, it is estimated that this period lasts three to six decades [8]. Even so, there is already some scientific evidence describing the association between cancer incidence and the occurrence of risk factors. According to the International Agency for Research on Cancer (IARC) [9], the carcinogens contributing to colorectal cancer with sufficient evidence in humans are as follows: alcoholic beverages; tobacco smoking; exposure to X-rays and gamma rays; and the consumption of processed meat. Other risk factors described in the literature include salt and salt-preserved food consumption, overweight and obesity, lack of physical activity [10] and low fruit and vegetable consumption [11]. Furthermore, fruit and vegetable consumption is indicated as a possible protective factor against the incidence of colorectal cancer [10].

In this context, our paper’s main goal is to describe and compare the geographical distribution of colorectal cancer incidence and mortality in mainland Portugal municipalities between 2007 and 2011. Although our purpose is not to describe the occurrence of risk factors in detail, they are considered in the discussion of the obtained results.

## Methods

**Fig. 1:**
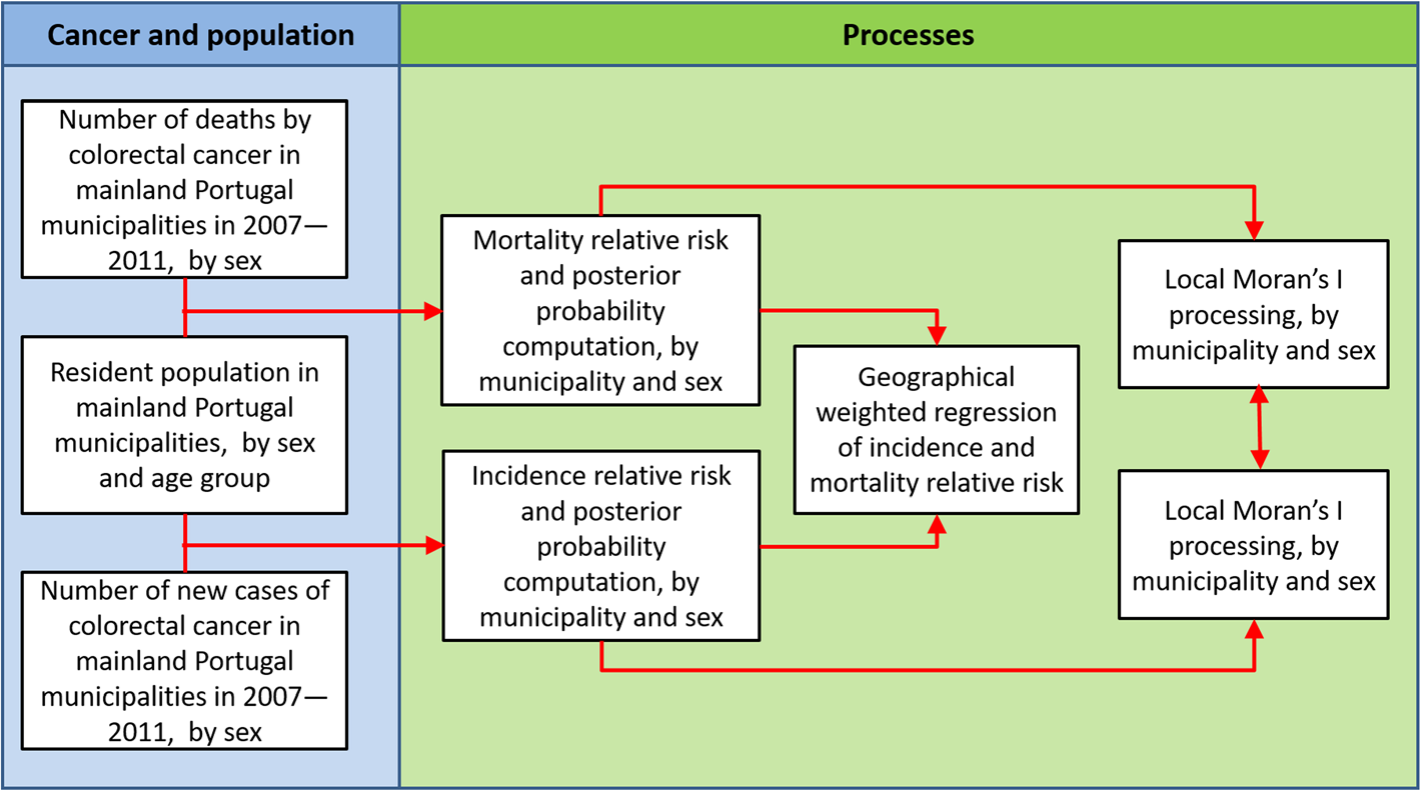
Methodological schema of data collection and geographical analysis

The flow diagram presented in Fig. 1 depicts the main methodological steps of our research, including the data sources and methods, which are described in the following sections.

### Base maps construction

A base map was constructed using Portugal’s official administrative cartography (CAOP) 2009 [12], corresponding to the central year of the analysis period. CAOP contains administrative limits at the smallest administrative level of Portugal, the parish. We applied a dissolve geoprocess to obtain more aggregated spatial limits, corresponding to municipalities.

Thus, another map with NUTS II (Nomenclature of territorial units for statistics) boundaries (a region composed of a set of municipalities) was constructed and overlaid on the maps of the municipalities. The names of each NUT II were indicated to facilitate the description of the results and the discussion. The NUTS II 1989 [13] boundary version was used for the discussion of risk factors. Unfortunately, there is no data for the risk factors available at the municipality level.

### Cancer and population data collection

New cancer cases were collected at the Regional Oncologic Registries (RORENO [14] from the North region, ROR Centro [15] from the Centre region and ROR Sul [16] from the South region). Mortality data were obtained from Statistics Portugal (INE) [17]. The codes considered for colorectal cancer were C18-C20, according to the 10^th^ revision of the International Statistical Classification of Diseases and Related Health Problems (10^th^ ICD) [18].

Data were disaggregated by 278 municipalities, according to sex and age groups (0–4, 5–9; 10–14; 15–19; 20–24; 25–29; 30–34; 35–39; 40–44; 45–49; 50–54; 55–59; 60–64; 65–69; 70–74; 75–79; ≥ 80 years old).

Due to the recommendation to use large populations and data grouping over several years [19], a 5-year period was adopted in the data analysis. On the other hand, because the last year of data on new cancer cases that were obtainable at the Portuguese Regional Oncological Registries (ROR) was 2011, the study period under consideration was 2007–2011.

Regarding the Portuguese population, we gathered data at INE for 2009, the central year of the study period, disaggregated by sex and the age groups previously described.

### Calculation of incidence and mortality relative risk and posterior probability

The Besag, York and Molié (BYM) model [20] was applied, using R-INLA [21], to estimate the relative risk (RR) of both colorectal cancer incidence and colorectal cancer mortality. The BYM is a Bayesian model that fits a Poisson spatial model, often used in spatial epidemiological studies [22], being suitable to analyse cancer data at the municipality level in mainland Portugal, in accordance with the findings of our previous research [23]. It applied observed data as dependent variables, expected data as offset variables and two random effects – spatial dependence and spatial heterogeneity – that solve the problem of over dispersion [24]. We adopt minimally informative priors: Besag model for spatial structured effect, with loggamma distribution of precision and parameters 1 and 0.001; iid model for unstructured effect, with loggamma distribution of precision and parameters 1 and 0.01.

In the model construction, spatial adjacency was considered as neighbourhood criterion, and “Laplace” was adopted as the strategy of integration. Expected cases were computed based on the mainland Portugal population in 2009, aggregated as described above and multiplied by 5 (years under analysis).

In result of model processing, we computed standardized incidence ratio and standardized mortality ratio (Additional file 1), the relative risk (RR) and the posterior probability (PP) of the RR being higher than 1.

The classes used by Mota and Falcão [25] were adopted for the legend of RR maps. The central class of the legend, indicated by the colour yellow, shows municipalities for which the RR is closer to the overall risk of mainland Portugal (RR = 1). Lower RRs were represented by the colour green and higher RRs with the colours orange and red.

The PP results were presented in maps constructed by an inverse distance weighted (IDW) interpolation process. The legends of the maps were classified by seven classes, created by the method of equal intervals.

### Local Moran’s index (LISA)

Through the ArcGIS program [26], LISA was applied to analyse the clusters of RR regarding both colorectal cancer incidence and colorectal cancer mortality. The parameters used in neighbourhood definition were the inverse distance and the Euclidean distance. The final maps were overlaid, which allowed us to find the municipalities belonging simultaneously to high-high cluster classes or low-low cluster classes in both the incidence and mortality maps.

### Geographically weighted regression (GWR)

We used GWR, performed in ArcGIS, to analyse the strength of association between colorectal cancer incidence and colorectal cancer mortality throughout mainland Portugal. GWR is a local regression method which applies an equation for each geographical unit under analysis.

Assuming that there is no underreport of the incidence and mortality rate, our hypothesis is that if the lethality rate of the individuals with colorectal cancer is equal between municipalities we would observe a proportionality between the incidence rate and the mortality rate. Meaning that the higher the incidence rate, the higher the mortality rate would be. A lack of fit of the model in certain areas would mean a deviance from this hypothesis and that there is no proportionality between incidence and mortality rate.

The RR concerning colorectal cancer mortality was assigned as a dependent variable, and the RR concerning colorectal cancer incidence was assigned as an independent variable. We assumed the adaptive kernel type and AICc bandwidth method.

## Results

Incidence and mortality have different distributions of RR values, as shown in Table 1. The interval of RR concerning incidence was higher than the interval of RR concerning mortality, among both men and women. Moreover, although mean values are close, standard deviation values are wider to incidence than to mortality.

**Table 1:** Statistics of RR regarding colorectal cancer incidence and mortality in mainland Portugal (2007–2011), by sex

		Number	Minimum	Maximum	Mean	Standard Deviation
Incidence	Men	22,143	0.19	4.79	0.95	0.35
	Women	15,400	0.19	2.50	0.91	0.29
Mortality	Men	10,245	0.84	1.19	1.00	0.08
	Women	7409	0.84	1.17	0.98	0.06

When comparing RR values between men and women, differences in RR values regarding incidence were found. The minimum RR value is the same (0.19), but the maximum RR value is higher among men than among women (4.79 and 2.50, respectively). This result is reflected in slightly greater mean values (0.95 and 0.91 for men and women, respectively) and a much higher standard deviation (0.35 to men and 0.29 to women). Mortality values, in turn, are very similar in both sexes.

In terms of the geographical distribution of the values, shown in Table 2, there are no large differences in RR mean values by NUT II in both incidence and mortality. Concerning incidence for both men and women, the highest mean RR values are found in the LVT region and the smallest in the Algarve region. Regarding mortality, the highest mean RR values are found in the Alentejo region for men and in the LVT region for women. The smallest RR values are found in the Norte region for both sexes.

**Table 2:** Statistics of RR regarding colorectal cancer incidence and mortality in mainland Portugal (2007–2011), by NUTSII

		Incidence					Mortality				
		Number	Min	Max	Mean	St. Dev.	Number	Min	Max	Mean	St. Dev.
Norte	Men	7547	0.19	1.90	0.93	0.28	3019	0.84	1.01	0.92	0.03
	Women	5375	0.19	1.75	0.91	0.30	2336	0.84	1.10	0.95	0.04
Centro	Men	5659	0.41	4.79	0.99	0.55	2738	0.91	1.10	0.99	0.04
	Women	3741	0.36	2.50	0.92	0.38	1930	0.89	1.07	0.96	0.04
LVT	Men	6101	0.32	1.45	1.00	0.19	2881	0.98	1.20	1.06	0.04
	Women	4470	0.25	1.38	0.96	0.22	2104	0.90	1.17	1.05	0.06
Alentejo	Men	1940	0.58	1.21	0.90	0.16	1147	1.01	1.18	1.09	0.04
	Women	1240	0.60	1.38	0.86	0.18	747	0.92	1.13	1.02	0.04
Algarve	Men	896	0.66	1.22	0.88	0.14	460	0.93	1.14	1.01	0.05
	Women	574	0.62	1.08	0.82	0.15	292	0.89	1.04	0.96	0.04

*Number* the number of new cases and number of deaths, *Min* Minimum, *Max* Maximum, *St. Dev* Standard Deviation

**Fig. 2:**
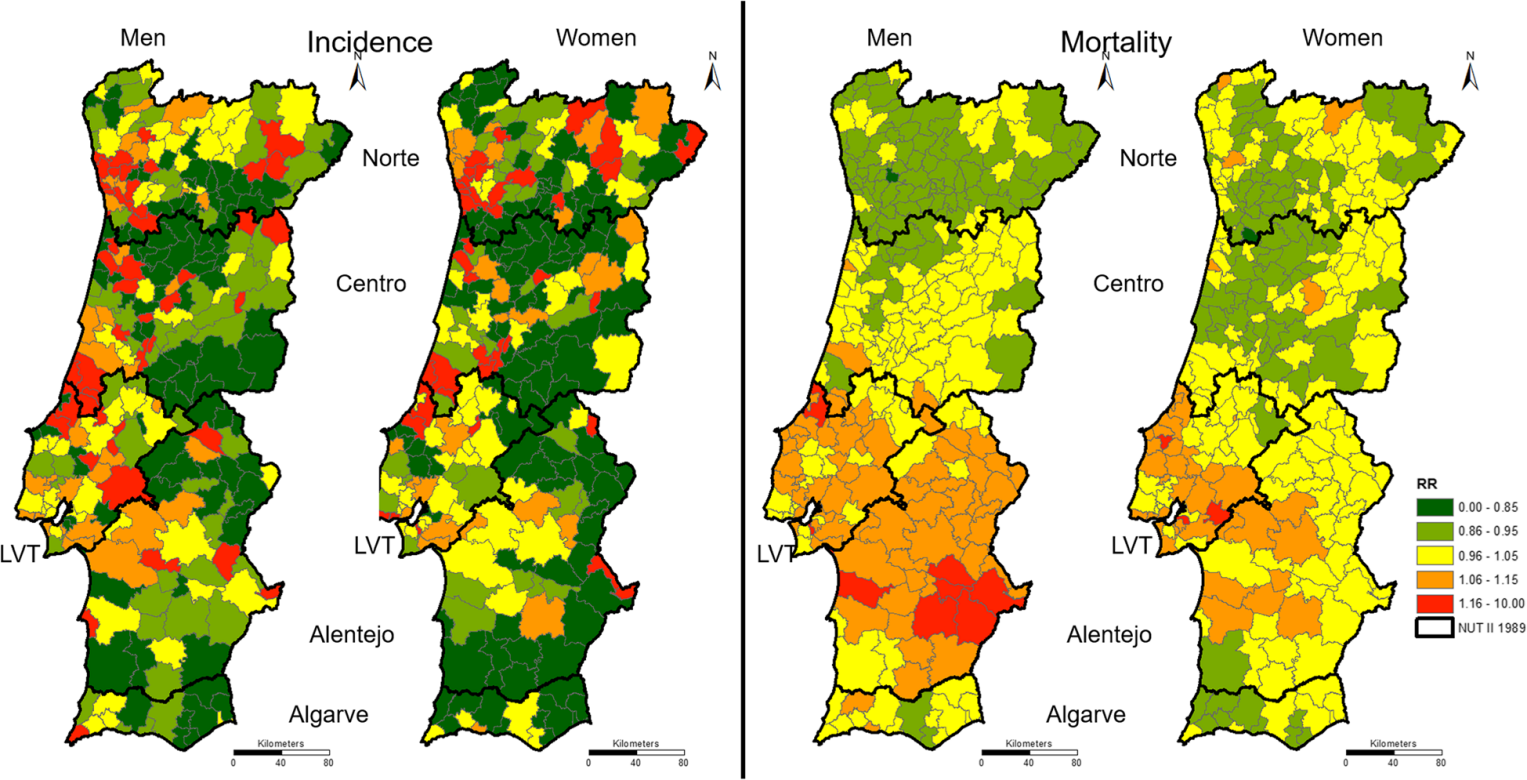
RR of colorectal cancer incidence (left maps) and mortality (right maps) in mainland Portugal, by sex

However, if we analyse the extreme values, different scenarios in incidence and mortality are found. The minimum RR values for mortality are always higher than the minimum RR values for incidence, and the maximum RR values for mortality are always smaller than the corresponding RR values for incidence in the same region. Moreover, the standard deviation of the RR values for mortality present low values, and they are very similar in all NUTS II and both sexes. These results are also shown in Fig. 2.

Overall, the incidence maps (at left) show that the RR of colorectal cancer incidence tends to be higher in the Norte region and the coastal municipalities of the Centro, LVT and Alentejo regions. However, there are differences in maps by sex. There are more municipalities with high RR values in the Norte region to women than to men. In turn, there are higher RR values in the coastal Centro, the LVT and, more markedly, the Alentejo region to men than to women.

In contrast, the mortality RR maps mostly present a more homogenous distribution of RR values in the entire territory than the incidence RR maps, with fewer municipalities classified in the extreme classes. The LVT and Alentejo regions, particularly, stand out as the regions with higher mortality RR values. These patterns are not as remarkable in the incidence RR maps.

**Fig. 3:**
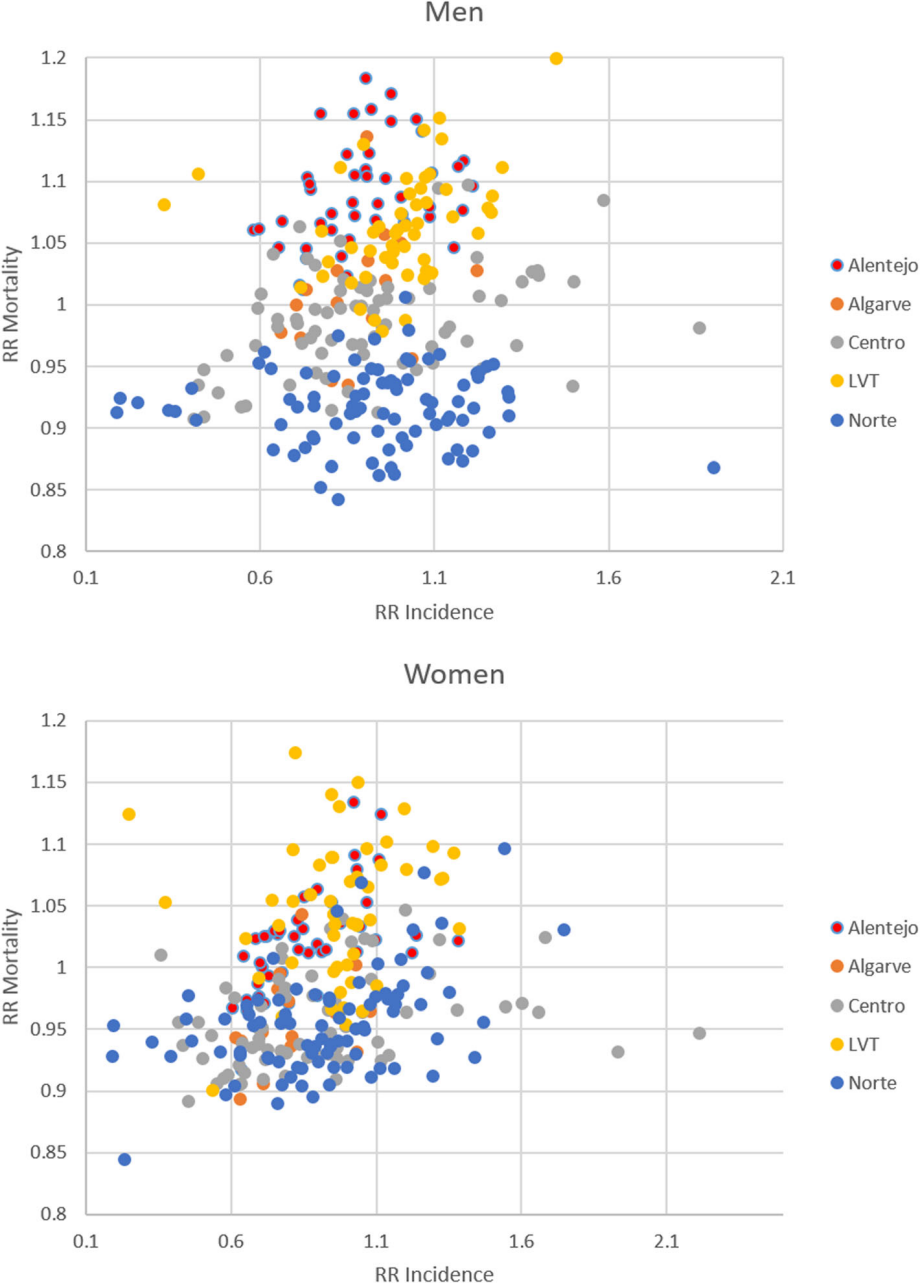
Scatterplot of RR incidence (X-axis) and mortality (Y-axis) by municipality, in men (top) and women (bottom). (legend) The colours attributed to the points correspond to the NUT II (1989) of each municipality

Figure 3 represents the values of the RR for incidence and mortality by municipality. Each point represents a municipality, coloured in the function of NUTS II. It was necessary to exclude two outliers with a very high incidence’s RR in the men’s graph to allow for the adequate visualisation of all the other points’ patterns.

One of the dominant patterns is the higher heterogeneity of RR incidence values in the Norte and Centro regions, particularly marked in the men’s scatterplot. On the other hand, the LVT and Norte regions are, at the same time, the regions with smaller RR mortality values.

Additionally, it is interesting to highlight that the few cases of low RR incidence in the LVT region simultaneously register high RR mortality.

In terms of RR mortality, for men, it is easy to identify Alentejo and, though less pronounced, LVT as the regions with higher values. These high values are, in general, associated with mean RR incidence values. In women, it is more difficult to highlight a region due to the greater heterogeneity in the distribution of values by region. However, it is apparent that some municipalities with higher RR mortality values are again associated with mean values for RR incidence.

**Fig. 4:**
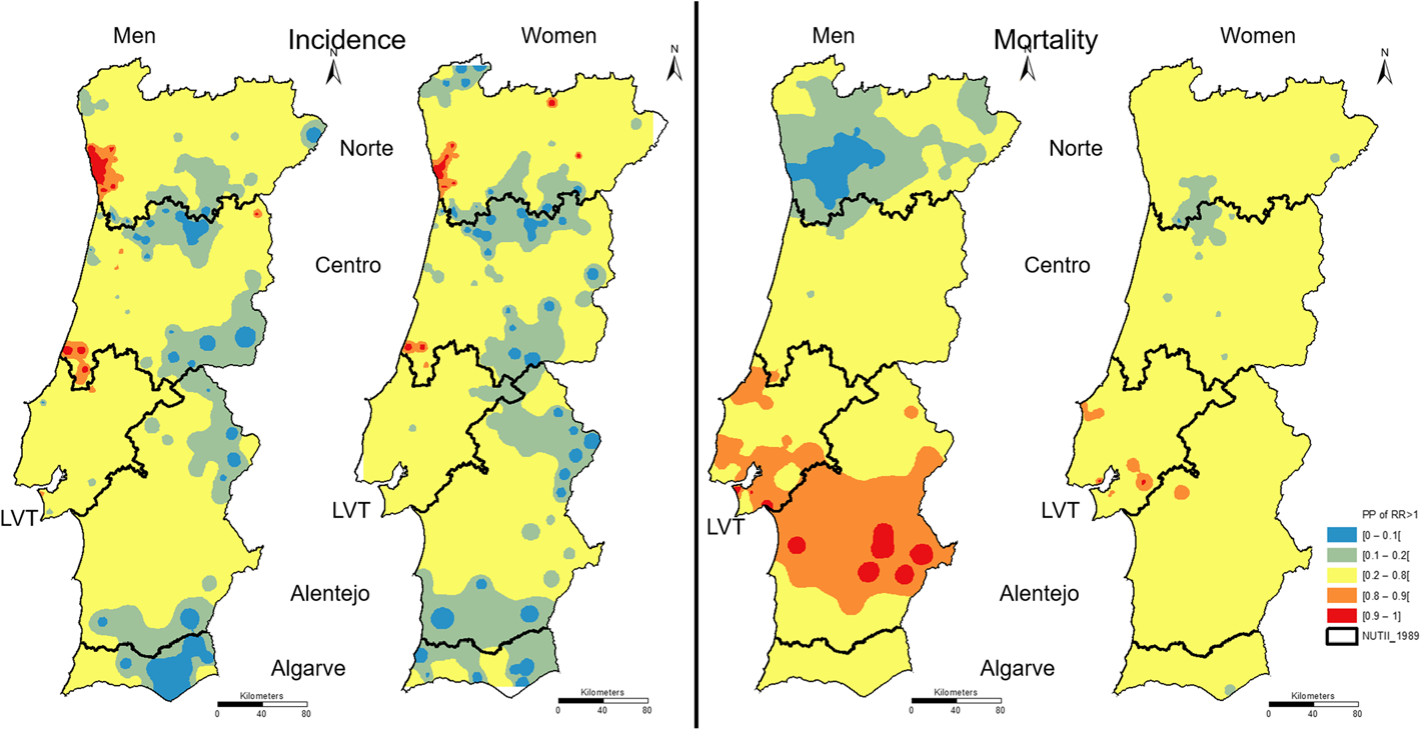
PP of RR of colorectal cancer incidence (left maps) and mortality (right maps) > 1

Concerning the situations in which the PP of the RR is higher than 1, the maps presented in Fig. 4 reinforce the finding of higher heterogeneity in the RR incidence of colorectal cancer than in the RR mortality of colorectal cancer. Incidence maps show high PP values agglomerating in the Norte and Centro coast. The high PP values in the incidence maps for the Norte and Centro regions are not sustained by high values of PP in the mortality maps. In turn, there is a much wider area of municipalities in the LVT and Alentejo regions to men.

**Fig. 5:**
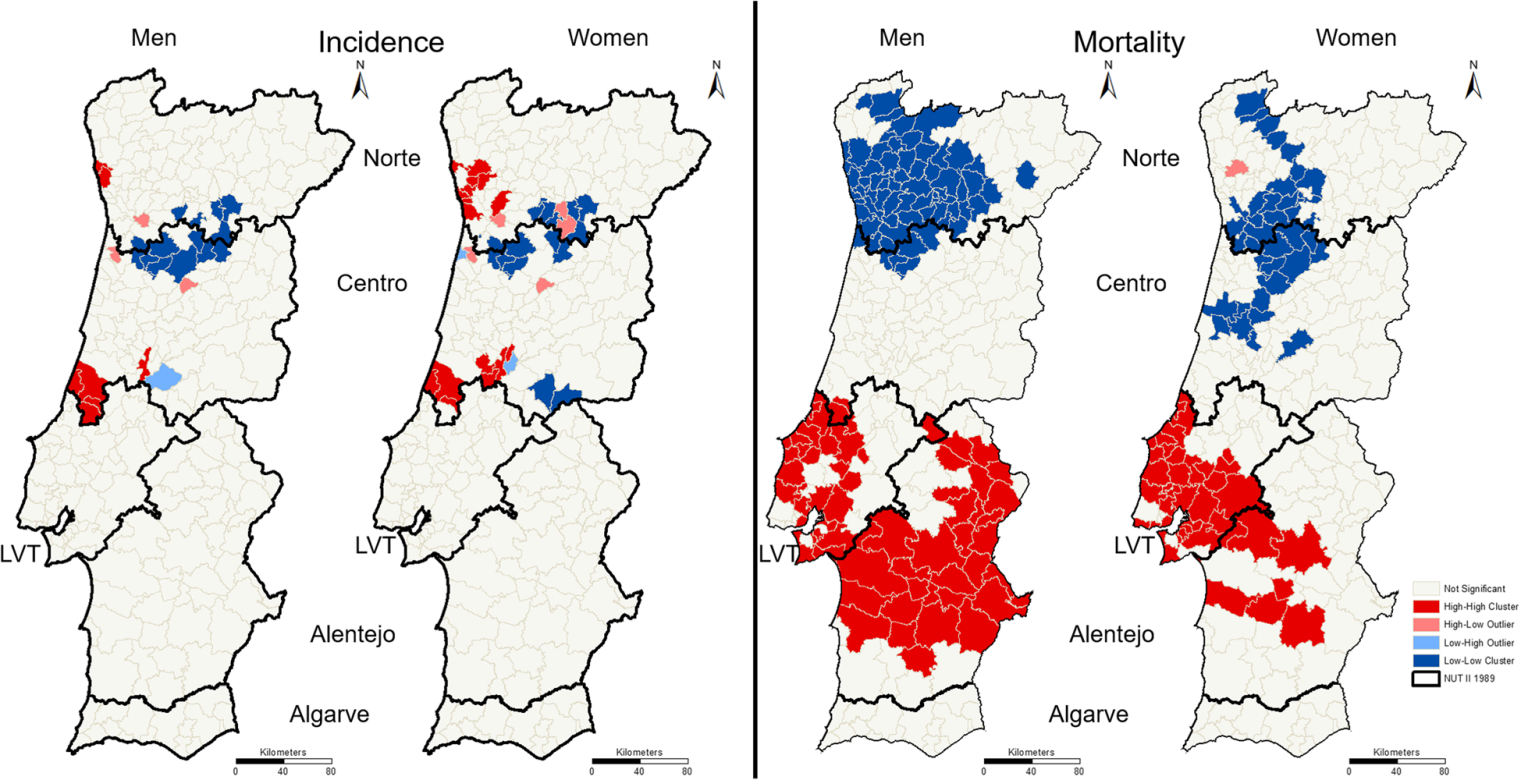
LISA of RR of colorectal cancer incidence (left maps) and mortality (right maps), by sex

LISA maps reveal much smaller clusters in incidence maps than in mortality maps (Fig. 5). In general, cluster maps reinforce the patterns presented in PP maps. Nevertheless, the clusters presented in LISA maps of incidence are smaller than in PP maps, and higher in mortality maps.

**Fig. 6:**
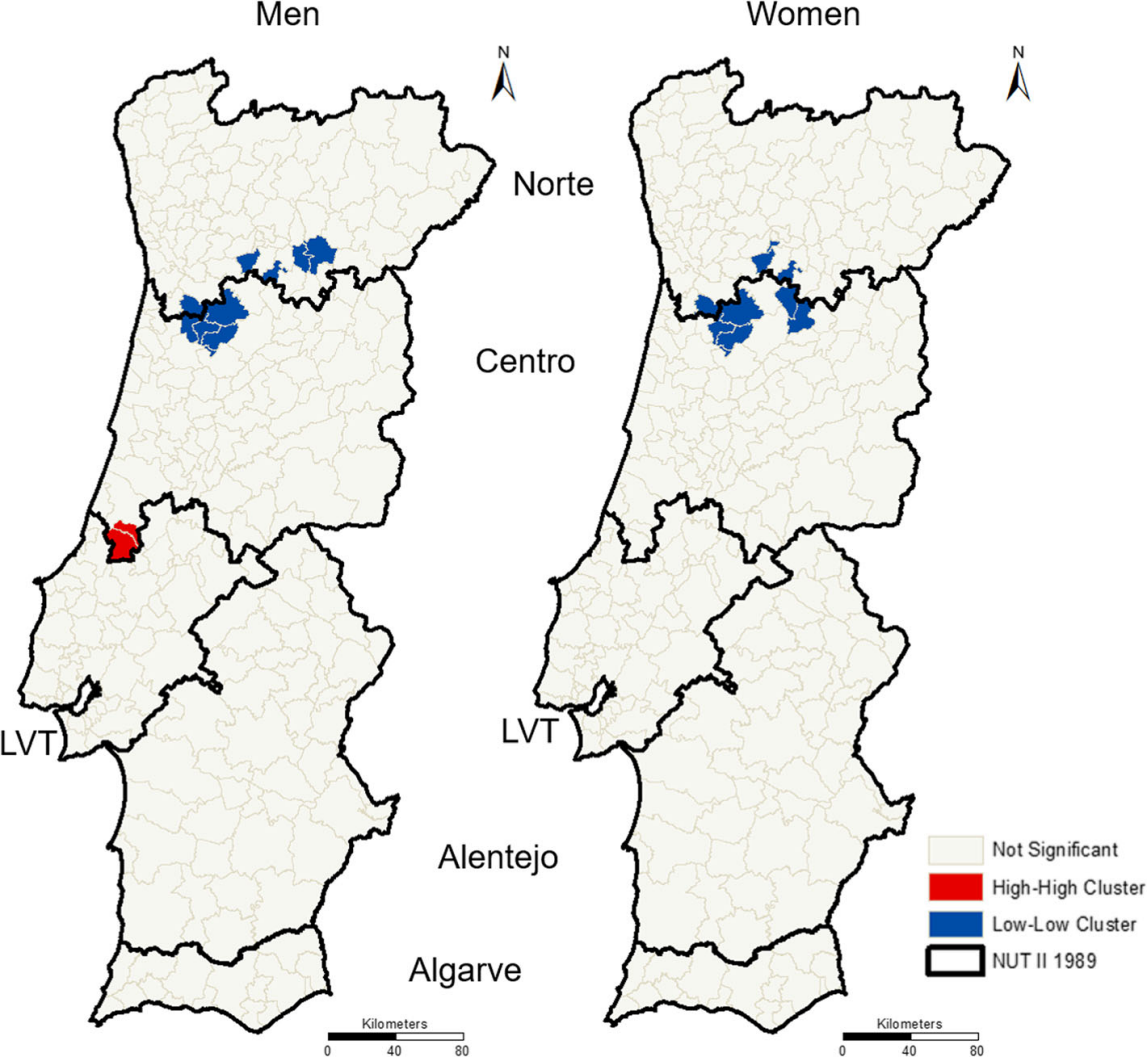
Municipalities in high-high or low-low clusters in both incidence and mortality maps, among men (left) and women (right)

In addition, we overlaid incidence and mortality LISA maps and looked up municipalities classified as low-low clusters and as high-high clusters in both maps, as presented in Fig. 6.

There are few municipalities classified in the same way in both the incidence and the mortality RR’s maps. For those municipalities, we emphasize two groups that deserve special attention: (1) Among men, the set of two municipalities belonging to the high-high cluster in incidence and mortality maps, situated in the boundary of the Centro and LVT regions; and (2) In both men and women, the set of 6 municipalities classified in the low-low cluster in incidence and mortality maps, located in the boundaries between the Norte and Centro regions.

**Fig. 7:**
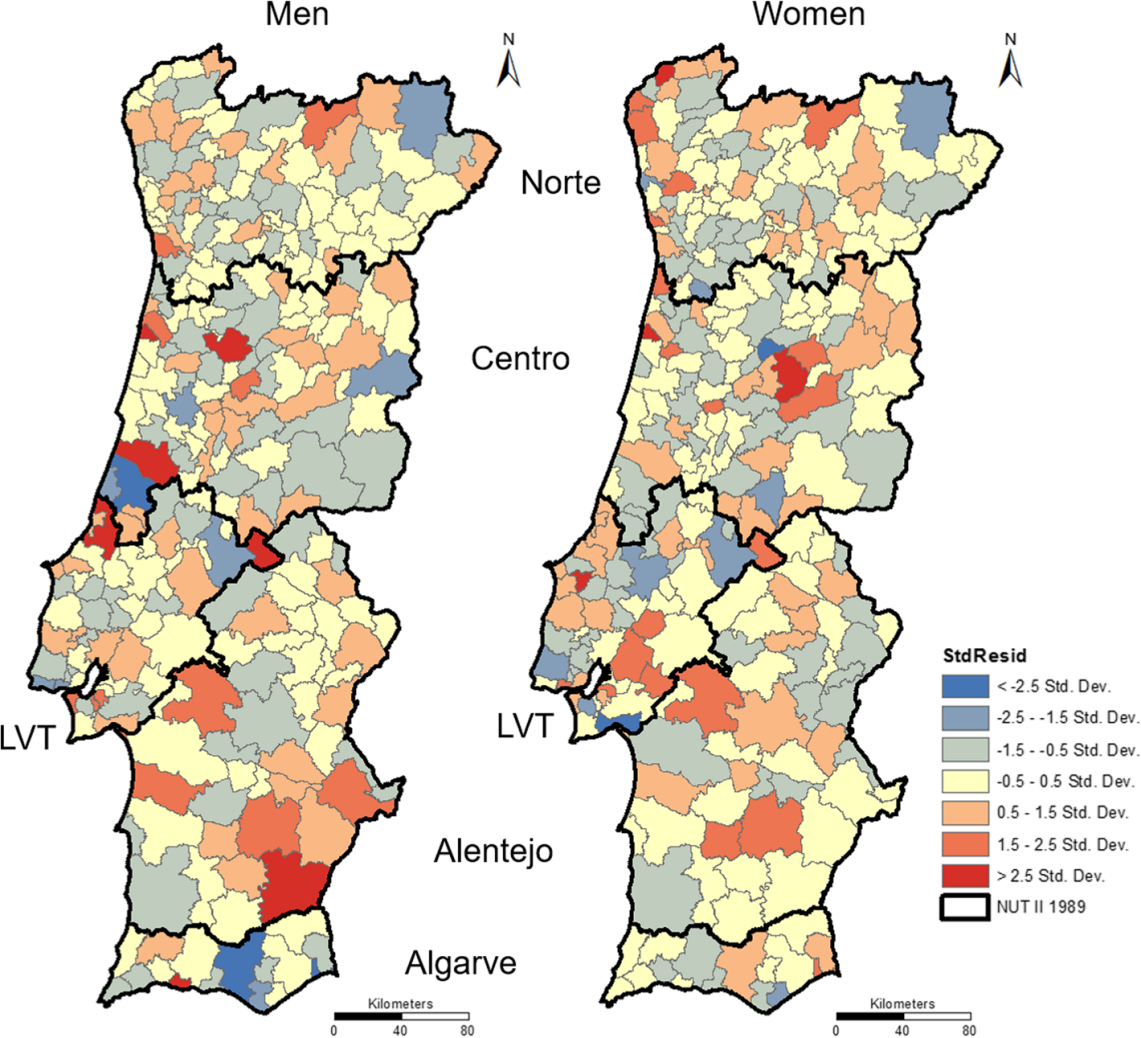
Standard residuals from GWR between incidence and mortality RR in men (left) and women (right)

To complement the comparison of RR incidence and RR mortality, we performed a GWR of these values for each sex. Residuals maps, presented in Fig. 7, show some municipalities with low and high values, particularly in the littoral Centro to men. However, with z values of − 0.46 and − 0.34, Moran I indicates a random distribution of these values, respectively for men and women.

**Fig. 8:**
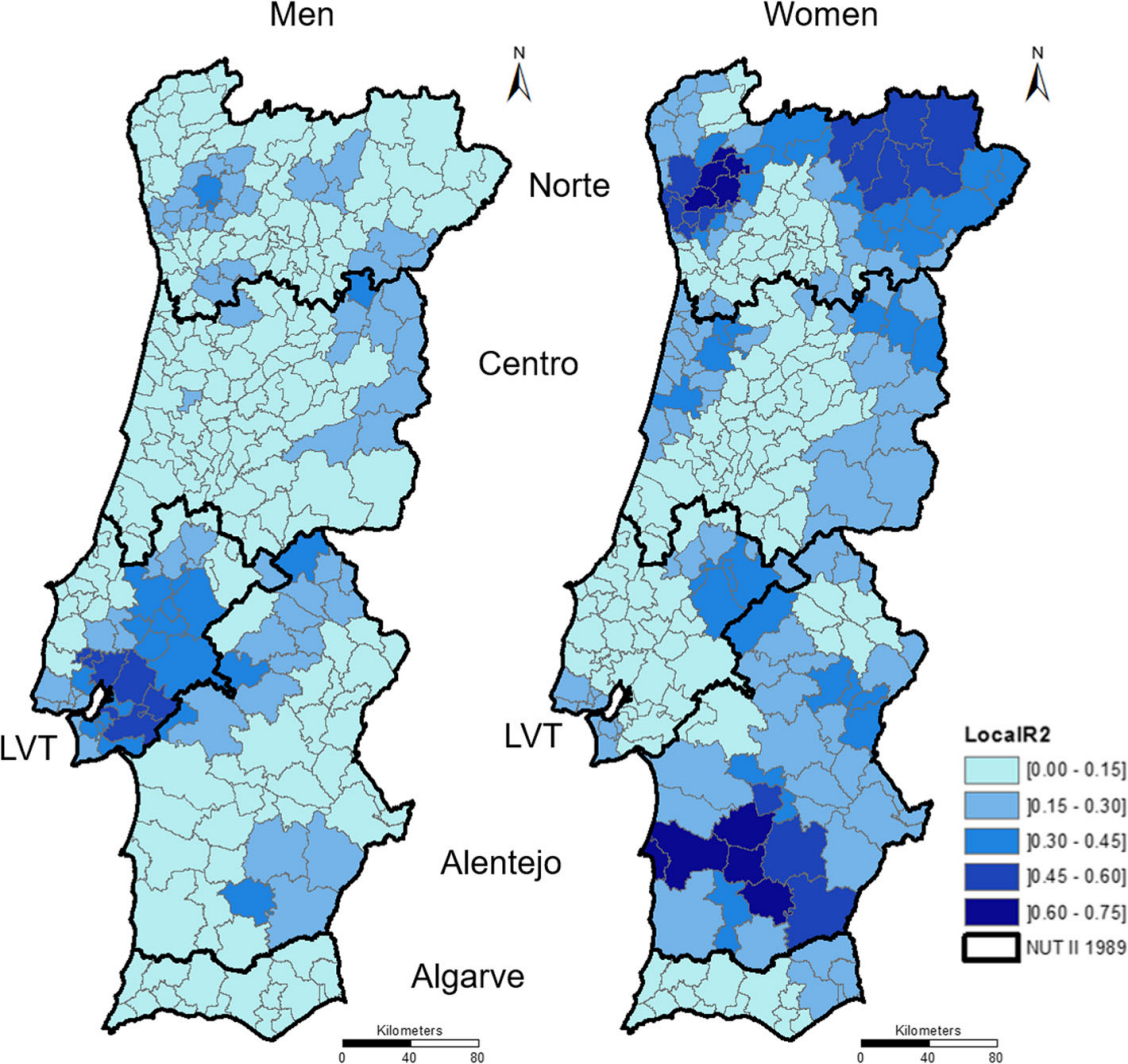
R^2^ from GWR between incidence RR and mortality RR in men (left) and women (right)

The R^2^ map classes show that the incidence RR values explain no more than 75% of the mortality RR values (Fig. 8). The highest values of R^2^ are located in the women’s map.

Referring to the men’s map, higher R^2^ values are found in the LVT region. Map patterns highlight other municipalities in the Alentejo, Norte and Centro regions but with lower values. In turn, in the women’s map, higher values are found in the Norte and Alentejo regions. However, all the other regions have municipalities with median values.

Table 3 reinforces previous findings. Moreover, the percentage of the RR mortality explained by the RR incidence is small for all regions and both sexes. This finding supports the differences in the RR incidence and RR mortality patterns.

**Table 3:** Statistics of R^2^ of GWR between colorectal cancer RR mortality and colorectal cancer RR incidence

		R^2^			
		Min	Max	Mean	St. Dev.
Norte	Men	0.00	0.33	0.09	0.10
	Women	0.00	0.73	0.26	0.19
Centro	Men	0.00	0.31	0.06	0.07
	Women	0.00	0.35	0.13	0.10
LVT	Men	0.00	0.59	0.26	0.17
	Women	0.00	0.42	0.12	0.12
Alentejo	Men	0.00	0.41	0.13	0.11
	Women	0.03	0.72	0.30	0.17
Algarve	Men	0.00	0.07	0.04	0.02
	Women	0.10	0.29	0.14	0.05

*Min* Minimum, *Max* Maximum, *St. Dev* Standard Deviation

In addition, the results in the table show that, in terms of means, the RR incidence explains a higher percentage of the RR mortality for women than for men in all NUTS II, with the exception of LVT.

## Discussion

Our results show differences in the geographical patterns of RR values between the incidence and mortality of colorectal cancer in mainland Portugal municipalities.

The distribution of RR values for incidence is more heterogeneous, registering both the lower and higher extreme values. The higher RR values for incidence are dominant in the Norte and Centro regions. These findings are reflected in the maps of the PP of the incidence RR > 1, where the high values are clustered on the West coast, particularly in the Norte, Centro and LVT regions. In the remaining territory, we identify only small areas with high values, surrounded by areas with lower values.

In turn, the distribution of the RR values of mortality is more homogeneous, with smaller variations in the extreme values. The RR mean values for mortality, which are higher than the RR mean values for incidence in some regions, are higher in the Alentejo region among men and in the LVT region among women. The RR mean values for mortality are smaller in the Norte region for both sexes. RR and PP maps show that higher values of mortality are clustered in the South of mainland Portugal, encompassing the Alentejo and LVT regions, with more expression among men than among women. These results are partially consistent with those of a previous study in which high values of mortality were identified for the Alentejo region in 2004 [5].

Regarding the comparison between men and women, we conclude that the RR values for the incidence and mortality of colorectal cancer are largest among men. Once more, this result is consistent with those of previously reported studies [2] [5].

The cluster maps analysis, presented in Fig. 5, reinforces the differences between the incidence and mortality geographical patterns. There are few municipalities represented in the same type of clusters (high-high or low-low) simultaneously in terms of the RR incidence and RR mortality (Fig. 6). It is particularly interesting to focus on these few municipalities and try to understand which factors justify the high values of incidence and mortality and which have a protective action and promote low values of incidence and mortality. This understanding can support the adoption of efficient measures in the fight against colorectal cancer incidence and mortality.

These results must be analysed considering the residuals’ map, presented in Fig. 7. Despite residuals are not very large in the majority of the areas, they form a cluster of extreme values (both high and low values) in the area classified with the cluster high-high in Fig. 6. This means that the relationship between incidence and mortality in these areas differs from other areas. This statement reinforces the need for more research in these geographical areas to evaluate why the relation between incidence and mortality is not similar to the majority of the municipalities. Among other hypothesis, this could mean that the lethality rate (survival) differs at municipality level, or that there is underreporting of the incidence or mortality rates.

Summing up, we found regional differences in colorectal cancer incidence and mortality risk. This statement is remarkable, especially since cancer’s mortality is related to cancer’s incidence and survival. Even though mortality not occur in the same year of the diagnosis, both the incidence and the mortality present a similar pattern of temporal evolution at mainland Portugal. Bearing this relationship in mind, we could expect that the spatial patterns of the incidence and the mortality were the same, but this is not the case. The results indicate that the RR of incidence explains, at most, 73% of the RR of mortality among women (in a municipality from the Norte region) and 59% among men (in a municipality from the LVT region) (Table 3). The municipalities where the RR of incidence better explains the RR of mortality are located in the LVT region for men and in the Norte and Alentejo regions for women.

One factor that plays an important role in incidence and mortality rates is screening. On the one hand, screening can be responsible for an increase in newly diagnosed cases [5] or over diagnosis, which occurs when the diagnosed cancer would be asymptomatic and never detected without the screening [27]. On the other hand, screening can contribute to early diagnosis and treatment, which will lead to higher survival rates [4].

In Portugal, organised screening for colorectal cancer has occurred since 2009. Screening guidelines define the target population as both men and women aged between 50 and 74 years old and recommend the use of the faecal occult blood test (FOBT) for all screening tests and colonoscopies for FOBT-positive cases [28].

There is a delay between the period of our analysis (2007–2011) and available data for screening (2015). This delay does not allow us to infer conclusions about colorectal cancer incidence or mortality and the occurrence of screening.

Another important issue is the future research that must be performed to compare the colorectal cancer incidence and mortality patterns with the main known colorectal cancer risk factors. As mentioned in the introduction section, there are several risk factors identified as potentially related to the occurrence of colorectal cancer.

In our study, we did not perform a detailed analysis of risk factors because the available data refer to a period too close to the time of collection of the cancer data, which does not allow us to consider the latency period. Moreover, the available data about referred risk factors have a high level of spatial aggregation. In fact, Portuguese national surveys adopt NUTS II or the Portuguese health regions as geographic units. It imposes the challenge of analysing data with different spatial scales, which is a common issue in spatial epidemiology [29]. Nevertheless, we performed a comparison of our results with the conclusions of some studies regarding some of the identified colorectal cancer risk factors.

One of the most important human carcinogens is tobacco smoking [30]. The results of the 4th Portuguese National Health Survey (4th INS) reveal that, in 2005, men smoke more than women in all NUTS II [31]. In mainland Portugal, the percentage of male smokers is highest in the Alentejo region, followed by the Algarve region. Among women, the highest percentages occur in the LVT and Algarve regions. These results are partially consistent with our PP (Fig. 4) and LISA maps (Fig. 5) when taking into account the different spatial aggregation levels. NUTS II with higher PP values (LVT and Alentejo regions) also register the highest percentage of smokers. The Algarve region is the only exception because the higher percentage of smokers is not followed by higher mortality or incidence PP values.

Another main colorectal cancer risk factor is alcohol intake. Alcohol consumption data from the 4th INS show that men drink more than women, regardless of age group or residence NUT II [31]. Furthermore, the percentage of the population who answered affirmatively about having drunk alcohol increased between the 3rd and 4th INS for both sexes.

Based on the 4th INS results [31], we verify that the region with the highest percentage of men consuming alcohol is Alentejo. Among women, the highest percentage belongs to the Norte region. These results are difficult to relate to the colorectal cancer incidence and mortality distribution in mainland Portugal municipalities. In fact, if we analyse alcohol intake among men, it seems to be in concordance with the LISA’s map of mortality, which presents a cluster of high values in the Alentejo region. However, when analysing the data on women, alcohol consumption seems to be more related to incidence maps, registering higher values in the Norte region. These results indicate the possible existence of modification effects promoted by other risk factors. For instance, some studies found a possible modification effect of alcohol consumption on the risk of colorectal cancer due to obesity [32].

Additionally, we eventually need another indicator to study the effect of alcohol consumption on colorectal cancer. One indicator related to alcohol intake is binge drinking, defined as the intake of six or more drinks during a single occasion. There is already evidence reporting that binge drinking may increase the risk of colorectal cancer [33]. Unfortunately, there is no data on binge drinking for our analysis period. Nevertheless, the data collected for 2015 show a high percentage of the Portuguese population of both sexes engaging in binge drinking in the Alentejo region [34]. Despite the time difference, these results seem to be consistent with our colorectal cancer mortality maps.

In terms of dietary behaviours, there is an association between colorectal cancer risk increasing and the following: a) a low consumption of certain foods such as soup, vegetables and fruit [35] and b) a high intake of red and processed meat [35] [33].

In the 4th INS, data were collected on vegetable and fruit consumption but not on processed meat consumption [31]. The results show that the population in mainland Portugal decreased the consumption of fruit and vegetables and increased the consumption of soup in all regions (with the exception of the Alentejo region for the latter) between 1998/1999 and 2005/2006. Moreover, the percentage of men who had eaten these types of foods was smaller than that of women for the same time periods and regions. These findings suggest a decrease in the consumption of foods that are indicated as having a potentially protective effect against colorectal cancer in all of mainland Portugal, independently of the region.

These data are not sufficient to characterise the dietary behaviours of the Portuguese population and their effect on colorectal cancer rates. More geographically detailed data are necessary to improve this analysis. Nevertheless, it seems that Portuguese dietary behaviours tend to be unhealthier. This finding is consistent with those of previous studies, in which changes in Portuguese dietary habits were demonstrated to be a contributor to the increase in the incidence of colorectal cancer [2].

In short, we would like to highlight some statements for each NUTS II that emerge from our findings:

- Norte has clusters of municipalities with a higher incidence of colorectal cancer. In terms of risk factor behaviours, it is important to be aware of the effects of smoking, alcohol intake (particularly in women) and the reduction in fruit and vegetable consumption;
- Centro registers the highest RR values for incidence. This situation merits investigation in the future to determine the reason underlying that occurrence. Regarding risk behaviours, smoking is increasing among women, and there is a reduction in fruit and vegetable consumption in both sexes;
- LVT stands out as one of the regions with a higher RR mortality, particularly among men. The risk factors that must be tracked are smoking and alcohol intake (particularly in women) and the reduction in fruit and vegetable consumption;
- Alentejo is the other region with a higher RR mortality, particularly among men. In this case, high mortality is accompanied by a low incidence of colorectal cancer. Regarding risk factors, it is important to highlight smoking, alcohol drinking (particularly in women) and the reduction in fruit, vegetable and soup consumption;
- Algarve has the lowest RR values for incidence. In turn, the RR values for mortality are not very high. Nevertheless, the increase in alcohol drinking (particularly in women) and the reduction in fruit and vegetable consumption are two behaviours that must be combatted.

## Conclusion

Colorectal cancer is an important public health concern in developed countries, where it predominantly affects people aged between 40 and 80 years old [36]. It is important to evaluate the patterns of both colorectal cancer and related geographical factors and, subsequently, use the knowledge generated in the measures for health planning. In fact, health policies and programmes must be based on evidence generated by the results of studies [37]. These results allow for the establishment of prevention measures, such as education, the promotion of healthy lifestyles or screening programmes [4].

Our findings show heterogeneous patterns in the distribution of colorectal cancer incidence and mortality in mainland Portugal municipalities during the period 2007–2011. Some of these patterns warrant more detailed study in the future, in order to understand the following:

- Why is incidence higher in some municipalities?
- Why is mortality not high in some municipalities where incidence is high?
- Finally, why is mortality high in municipalities where incidence is not high?

To answer these questions, it is necessary to analyse risk factors as well as the availability and performance of health services.

The evaluation of risk factors was not the aim of our research. Nevertheless, we highlight situations that can provide clues regarding more detailed studies in the future, specifically including the following: a) the possibility of a relation between opportunistic screening and high incidence; b) the high prevalence of smoking and alcoholic beverage intake; and c) the adoption of unhealthier dietary habits. Furthermore, we correlate the RR values for incidence with those for mortality. The results of this analysis allow for the identification of the municipalities where the incidence values poorly explain the mortality values. In those municipalities, it is possible that some risk factors contribute to a higher lethality of colorectal cancer.

Furthermore, it is relevant to highlight the small cluster of municipalities that register high values for both incidence and mortality (Fig. 5). It is important to understand why the incidence of colorectal cancer is higher in these municipalities. In addition, it is important to see if the high mortality is a natural consequence of the high incidence. Alternatively, for other hypotheses, it is relevant to understand if there is a higher lethality of colorectal cancer in those municipalities and, if so, to try to identify the factors that can explain this finding.

To finish, we would like to reiterate that the fight against risk factors is the key to decreasing cancer morbidity and mortality [38]. Knowledge of the geographical patterns of cancer incidence and mortality and the development of healthy behaviours are essential for that fight.

## Additional file

Additional file 1: SIR and SMR of colorectal cancer in mainland Portugal, by sex. (ZIP 1260 kb)

## Abbreviations

BYM: Besag, York and Mollié model
CAOP: Official Administrative Map of Portugal
GWR: Geographically weighted regression
ICD 10th: 10th revision of International Statistical Classification of Diseases and Related Health
IDW: Inverse distance weighted
INE: Statistics Portugal
INS: National Health Survey
LISA: Local Moran’s I
Moran I: Global Moran’s I
NUTS II: Nomenclature of Territorial Units for Statistics (level II)
PP: Posterior probability
ROR: Regional Oncological Registries
RR: Relative risk
SMR: Standardized Mortality Ratio

## References

[1] Karim-Kos HE, de Vries E, Soerjomataram I, Lemmens V, Siesling S, Coebergh JWW (2008). Recent trends of cancer in Europe: a combined approach of incidence, survival and mortality for 17 cancer sites since the 1990s. Eur J Cancer.

[2] Cotter J (2013). Colorectal cancer: Portugal and the world. Acta Medica Port.

[3] Bosetti C, Levi F, Rosato V, Bertuccio P, Lucchini F, Negri E, La Vecchia C (2011). Recent trends in colorectal cancer mortality in Europe. Int J Cancer.

[4] Pinheiro P, Tyczynski J, Bray F, Amado J, Matos E, Miranda A, Limbert E. Cancer in Portugal. In: IARC, editor. IARC technical publication no 38. France, IARC, 2002. p. 72.

[5] Pinto CG, Paquete AT, Pissarra I (2010). Colorectal cancer in Portugal. Eur J Health Econ.

[6] Miranda N, Portugal C, Nogueira PJ, Farinha CS, Oliveira AL, Alves MI, Martins J (2016). Portugal Doenças Oncológicas em números, 2015.

[7] NCI dictionary of cancer terms [https://www.cancer.gov/publications/dictionaries/cancer-terms?expand=L], Accessed 30 Oct 2017.

[8] Nadler Diana L., Zurbenko Igor G. (2014). Estimating Cancer Latency Times Using a Weibull Model. Advances in Epidemiology.

[9] IARC: List of classification by cancer sites with sufficuent or limited evidence in humans, volumes 1 to 120. France: IARC; 2017.

[10] NIH (2017). Cancer and the environment.

[11] Lock K, Pomerleau J, Causer L, McKee M. Low fruit and vegetable consumption. In: Ezzati M, Lopez AD, Rodgers A, Murray CJ, editors. Comparative quantification of health risks. Global and regional burden of disease attributable to selected major risk factors. Geneva: World Health Organization; 2004. p. 597-728.

[12] Carta Administrativa Oficial de Portugal (CAOP) Versão 2009. http://www.dgterritorio.pt/dados_abertos/caop/. Accessed 15 May 2017.

[13] Decreto Lei 46/89 de 15 de Fevereiro In *Diário da república n°38/89, I série*. Lisboa: Ministério do Planeamento e da Administração do Território; 1989.

[14] RORENO [http://www.roreno.com.pt/]. Accessed 30 Oct 2017.

[15] ROR Centro [https://ipocoimbra.com/ror/]. Accessed 30 Oct 2017.

[16] ROR Sul [https://ron.min-saude.pt/pt/]. Accessed 30 Oct 2017.

[17] Statistics Portugal [https://ine.pt/xportal/xmain?xpgid=ine_main&xpid=INE]. Accessed 30 Oct 2017.

[18] WHO. International statistical classification of diseases and related health problems - 10th revision. Geneve: World Health Organization; 2016.

[19] Jensen OM. Cancer registration: principles and methods. France: IARC; 1991. 1894317

[20] Besag J, York J, Mollié A (1991). Bayesian image restoration, with two applications in spatial statistics. Ann Inst Stat Math.

[21] Rue H, Martino S, Chopin N (2009). Approximate Bayesian inference for latent Gaussian models by using integrated nested Laplace approximations. J R Stat Soc Ser B Stat Methodol.

[22] Blangiardo M, Cameletti M (2015). Spatial and spatio-temporal Bayesian models with R-INLA.

[23] Roquette R, Nunes B, Painho M (2018). The relevance of spatial aggregation level and of applied methods in the analysis of geographical distribution of cancer mortality in mainland Portugal (2009–2013). Popul Health Metrics.

[24] Lopez-Abente G, Aragones N, Perez-Gomez B, Pollan M, Garcia-Perez J, Ramis R, Fernandez-Navarro P (2014). Time trends in municipal distribution patterns of cancer mortality in Spain. BMC Cancer..

[25] Mota L, Falcão J (1997). 2° Atlas da mortalidade por cancro em Portugal 1990–1992. Lisboa: Departamento de Estudos e Planeamento da Saúde.

[26] ArcGIS [https://www.arcgis.com/features/index.html], Accessed 10 Mar 2018.

[27] Welch HG, Black WC (2010). Overdiagnosis in cancer. J Natl Cancer Inst.

[28] DGS (2016). Programa Nacional para as Doenças Oncológicas. Avaliação e Monitorização dos Rastreios Oncológicos Organizados de Base Populacional de Portugal. Relatório.

[29] Saib M-S, Caudeville J, Carre F, Ganry O, Trugeon A, Cicolella A (2014). Spatial relationship quantification between environmental, socioeconomic and health data at different geographic levels. Int J Environ Res Public Health.

[30] Lagiou P, Trichopoulou A, Trichopoulos D (2002). Nutritional epidemiology of cancer: accomplishments and prospects. Proc Nutr Soc.

[31] INSA/INE (2009). Inquérito Nacional de Saúde 2005/2006.

[32] Zhao J, Zhu Y, Wang PP, West R, Buehler S, Sun Z, Squires J, Roebothan B, McLaughlin JR, Campbell PT (2012). Interaction between alcohol drinking and obesity in relation to colorectal cancer risk: a case-control study in Newfoundland and Labrador, Canada. BMC Public Health.

[33] Stewart BW, Kleihues P. World cancer report. France: IARC; 2003.

[34] INSA: 1° Inquérito Nacional de Saúde com Exame Físico (INSEF 2015): Determinantes de Saúde. Lisboa: Instituto Nacional de Saúde Doutor Ricardo Jorge, IP; 2017.

[35] Magalhães B, Bastos J, Lunet N (2011). Dietary patterns and colorectal cancer: a case–control study from Portugal. Eur J Cancer Prev.

[36] Rougier P, Mitry E (2003). Epidemiology, treatment and chemoprevention in colorectal cancer. Ann Oncol.

[37] Ott JJ, Ullrich A, Mascarenhas M, Stevens GA (2011). Global cancer incidence and mortality caused by behavior and infection. J Public Health.

[38] Danaei G, Vander Hoorn S, Lopez AD, Murray CJ, Ezzati M (2005). Group CRAc: causes of cancer in the world: comparative risk assessment of nine behavioural and environmental risk factors. Lancet.

