# Supplementary figures and images for: Geographical patterns of the incidence and mortality of colorectal cancer in mainland Portugal municipalities (2007–2011)
Source: BMC Cancer. 2019 May 29;19:512. doi: 10.1186/s12885-019-5719-9 (PMC6542026; doi:10.1186/s12885-019-5719-9)

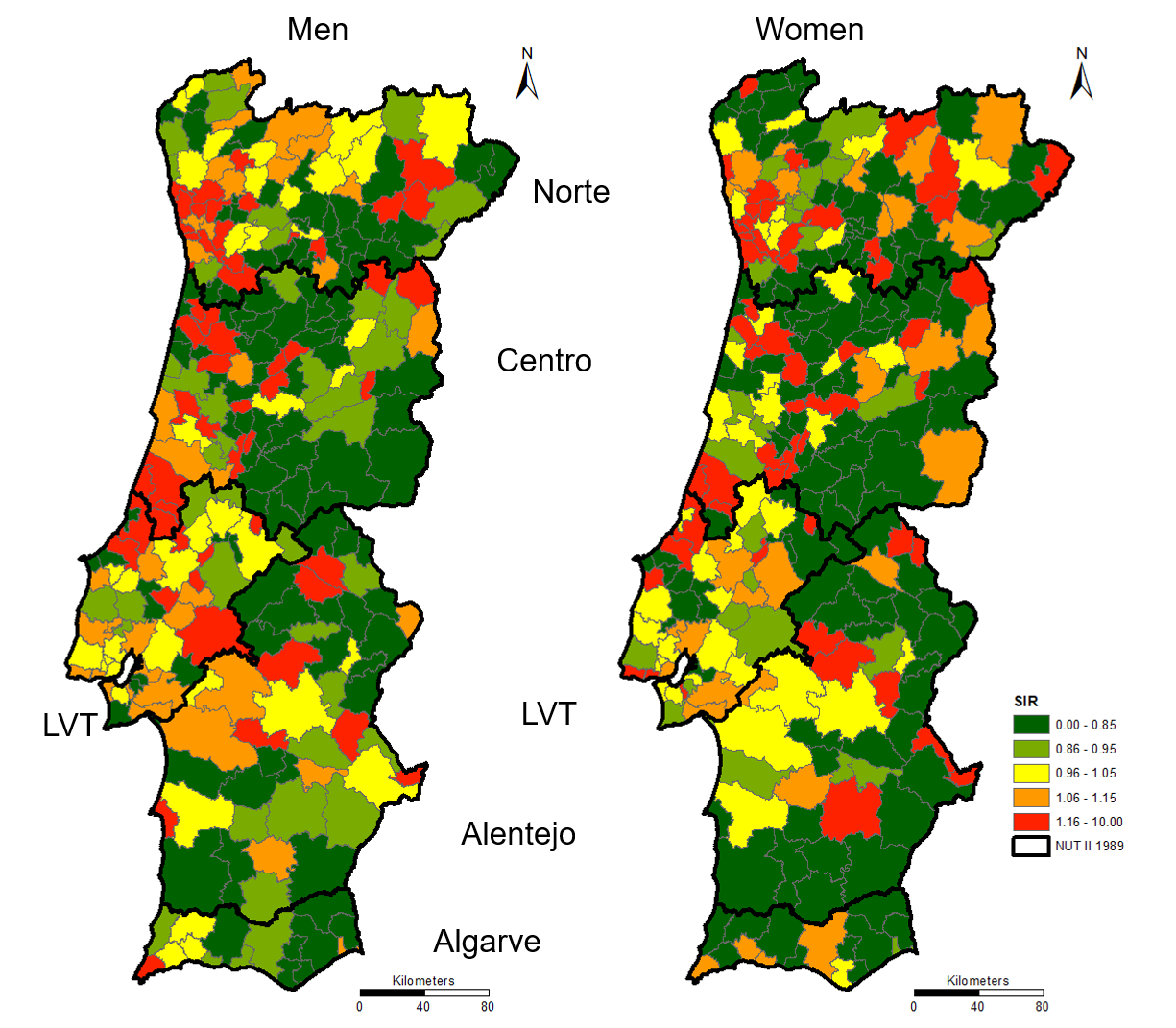

Supplement: Supplementary file 1 — SIR and SMR of colorectal cancer in mainland Portugal, by sex. (ZIP 1260 kb) [file 12885_2019_5719_MOESM1_ESM.zip › Figura_anexo_SIRR4.tif]

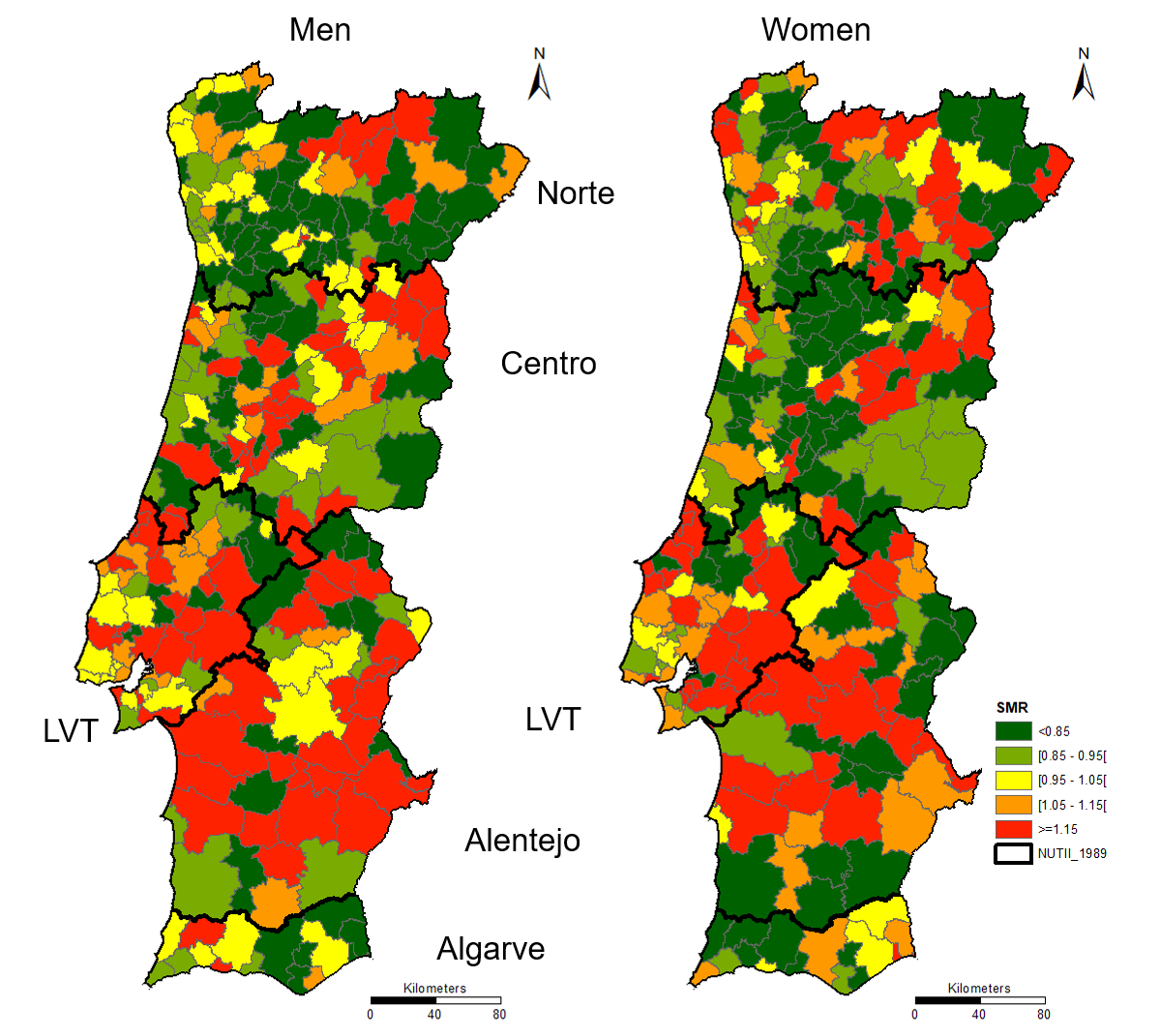

Supplement: Supplementary file 1 — SIR and SMR of colorectal cancer in mainland Portugal, by sex. (ZIP 1260 kb) [file 12885_2019_5719_MOESM1_ESM.zip › Figura_anexo_SMRR4.tif]
